# Multicomponent Domino Synthesis, Anticancer Activity and Molecular Modeling Simulation of Complex Dispirooxindolopyrrolidines

**DOI:** 10.3390/molecules23051094

**Published:** 2018-05-05

**Authors:** Natarajan Arumugam, Abdulrahman I. Almansour, Raju Suresh Kumar, Periyasami Govindasami, Dhaifallah M. Al-thamili, Rajapandian Krishnamoorthy, Vaiyapuri Subbarayan Periasamy, Ali A. Alshatwi, S. M. Mahalingam, Shankar Thangamani, J. Carlos Menéndez

**Affiliations:** 1 Department of Chemistry, College of Science, King Saud University, P.O. Box 2455, Riyadh 11451, Saudi Arabia; almansor@ksu.edu.sa (A.I.A.); sraju@ksu.edu.sa (R.S.K.); pkandhan@ksu.edu.sa (P.G.); daife54321@hotmail.com (D.M.A.); 2 Nanobiotecnology and Molecular Biology Research Laboratory, Department of Food Science and Nutrition, College of Food and Agricultural Sciences, King Saud University, Riyadh 11451, Saudi Arabia; rjpandiyank@gmail.com (R.K.); alshatwi@ksu.edu.sa (A.A.A.); 3 Department of Chemistry, Purdue University, 560 Oval Drive, West Lafayette, IN 47907-2084, USA; vsperrys@gmail.com (V.S.P.); mahalins@purdue.edu (S.M.M.); 4 Department of Pathology and Population Medicine, College of Veterinary Medicine, Midwestern University, Glendale, AZ 85308, USA; sthang@midwestern.edu; 5 Unidad de Química Orgánica y Farmacéutica, Departamento de Química en Ciencias Farmacéuticas, Facultad de Farmacia, Universidad Complutense, 28040 Madrid, Spain

**Keywords:** domino reaction, 1,3-dipolar cycloaddition, ionic liquids, cytotoxicity assays, molecular docking

## Abstract

A series of spirooxindolopyrrolidine fused *N*-styrylpiperidone heterocyclic hybrids has been synthesized in excellent yield via a domino multicomponent protocol that involves one-pot three component 1,3-dipolar cycloaddition and concomitant enamine reactions performed in an inexpensive ionic liquid, namely 1-butyl-3-methylimidazolium bromide ([bmim]Br). Compounds thus synthesized were evaluated for their cytotoxicity against U-937 tumor cells. Interestingly; compounds **5i** and **5m** exhibited a better cytotoxicity than the anticancer drug bleomycin. In addition; the effect of the synthesized compounds on the nuclear morphology of U937 FaDu cells revealed that treatment with compounds **5a**–**m** led to their apoptotic cell death.

## 1. Introduction

The word “cancer” refers to a potentially lethal group of diseases characterized by unregulated proliferation and a dysregulation of apoptotic mechanisms. The development of resistance to chemotherapeutic agents and associated side effects are major obstacles to treat cancer effectively [1]. Thus, it is necessary to identify and develop new anti-cancer agents with improved efficacy and reduced side effects to complement the present chemotherapeutic strategies.

Apoptosis, an orchestrated event in which cells are programmed to die in response to specific stimuli, is crucial for sustaining the physiologic balance between cell growth and cell death [2]. Apoptosis can be triggered by two major pathways: the extrinsic pathway that involves binding of “death ligands” to “death receptors” and the intrinsic mitochondrial pathway [3], initiated by cytotoxicity, and both pathways are regulated by a group of proteases known as caspases [4]. Targeting these pathways by chemotherapeutic drugs is a proven therapeutic strategy to control tumor growth and cancer progression [5].

Medicinal chemistry is devoted to the challenging task of designing new synthetic compounds with therapeutic importance. In particular, spirooxindole-pyrrolidine ring systems are a privileged class of heterocycles, as shown by their prevalence in several natural alkaloids such as spirotryprostatins A and B [6], horsfiline [7], elacomine [8], formosanine [9] and rhynchophylline [10] that possesses many biological activities including antimicrobial and antitumor properties against brain cancer cell lines, neuroblastoma SKN-BE and malignant glioma GAMG [11].

**Figure 1:**
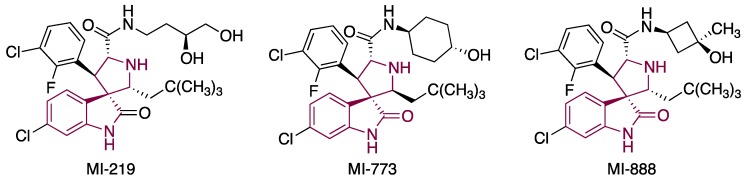
Some spiro(oxindole-3,3′-pyrrolidine) derivatives that inhibit the mdm2–p53 interaction.

Some synthetic spiro(oxindole-pyrrolidine) derivatives that are relevant to the work described here are shown in Figure 1. These compounds interfere with the proteasomal degradation of p53, a transcription factor known as the “guardian of the human genome” that controls the induction of apoptosis by several kinds of stress and is mutated or deleted in about 55% of human cancers. One of the mechanisms that regulates the function of p53 is its degradation by the proteasome, which requires its prior interaction with mdm2, an ubiquitin ligase. For this reason, compounds able to antagonize the p53/mdm2 interaction hold a great potential as anticancer agents [12]. In this connection, the spiro(oxindole-3,3′-pyrrolidine) derivatives MI-219, MI-773, MI-888 and related compounds are very promising because they show a strong affinity for the pocket of mdm2 where the α-helix of p53 is attached when both proteins interact. To this core structure, we have added a bis-arylidinepiperidone unit because some derivatives of this system have displayed excellent anti-cancer activities [13,14,15].

Despite their potential biological significance, the synthesis of spirooxindole-pyrrolidines containing a bis-arylidenene piperidone moiety has received little attention. In the context of our research in the field of domino processes based on 1,3-dipolar cycloadditions [16,17,18,19,20,21,22], herein we report an easy access to hybrid heterocycles formed by a spirooxindole-pyrrolidine unit attached to a *N*-styrylpiperidone moiety via a one-pot, green synthetic protocol in an ionic liquid, together with their biological evaluation.

## 2. Results and Discussion

### 2.1. Chemistry

**Scheme 1:**
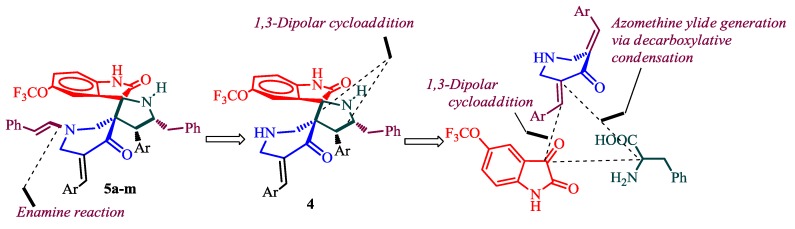
The synthetic strategy for the preparation of spiropyrrolidine/*N*-styrylpiperidone heterocyclic hybrids.

**Scheme 2:**
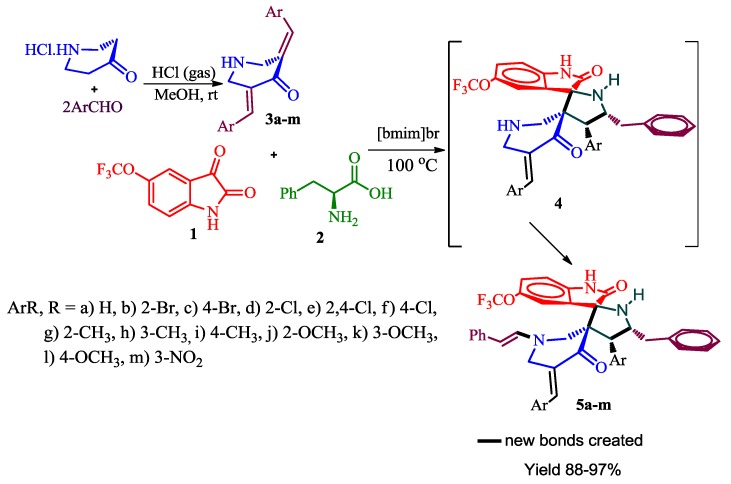
Synthesis of dispirooxindolopyrrolidine fused *N*-styrylpiperidone heterocyclic hybrids.

Our methodology was based on the multicomponent dipolar cycloaddition reaction strategy described in Scheme 1, which involves a domino process performed in an ionic liquid, 1-butyl-3-methylimidazolium bromide ([bmim]Br) at 100 °C, and centered around a 1,3-dipolar cycloaddition reaction between (3*E*,5*E*)-3,5-bis(4-methylbenzylidene)piperidin-4-one [16] and a rare class of azomethine ylides **6**, generated in situ from 5-(trifluoromethoxy)isatin (**1**) and l-phenylalanine (**2**). The spirohybrid heterocycle **4** thus generated subsequently reacted with phenylacetaldehyde (**8**) generated in situ from azomethine ylide **6**, to afford the unusual dispiropyrrolidine tethered *N*-styrylpiperidone heterocyclic hybrids **5** through an enamine formation reaction (Scheme 2).

For optimization, the reaction was initially performed in methanol starting from (3*E*,5*E*)-3,5-bis(4-methylbenzylidene)piperidin-4-one (**3i**, 1 equiv), 5-(trifluoromethoxy)isatin (**1**, 2 equiv) and l-phenylalanine (**2**, 2.05 equiv), affording the product **5i** in good yield. In a move towards an environmentally begin synthesis, this domino reaction was performed in different ionic liquids *viz*., [bmim]OH, [bmim]BF_4_, [bmim]PF_6_ and [bmim]Br at 100 °C for 1 h. Among them, [bmim]Br afforded the highest yield of the product, as a single regioisomer and in a shorter reaction time (Table 1). Consequently, all the subsequent reactions were performed under these optimized conditions.

**Figure 2:**
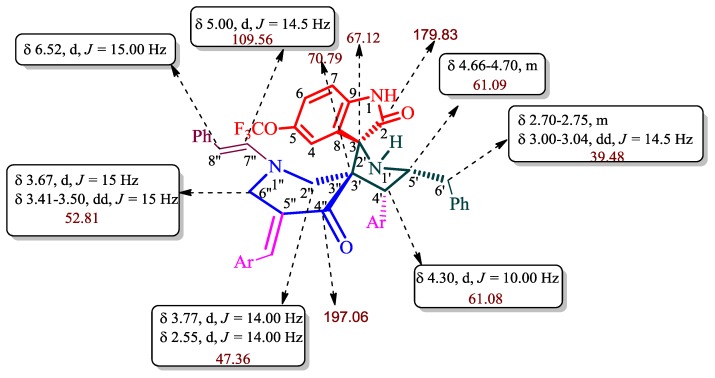
Selected chemical shift values of **5i**.

**Figure 3:**
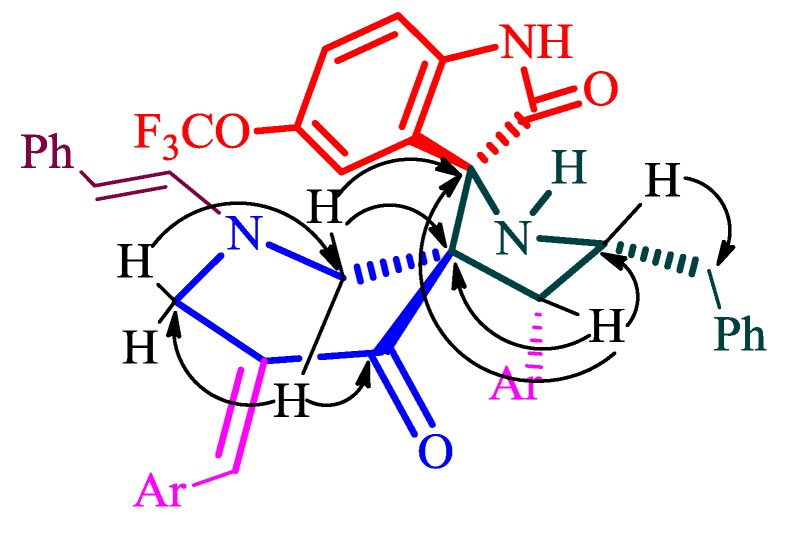
Selected HMBC correlations of **5i**.

**Figure 4:**
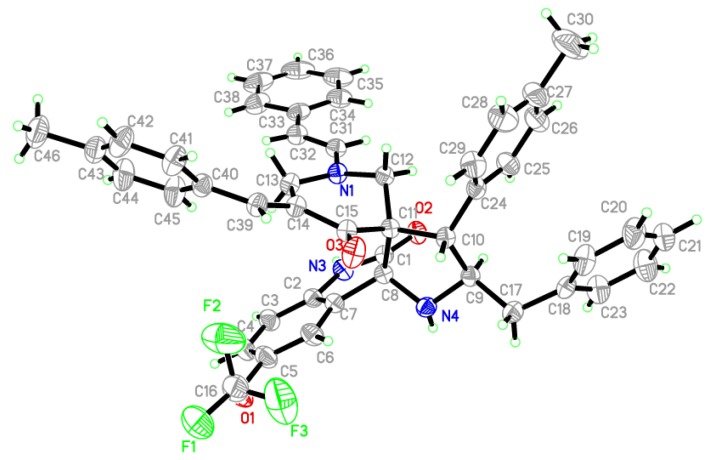
Oak Ridge Thermal-Ellipsoid Plot (ORTEP) of spiroheterocyclic hybrid, **5i**.

The structural elucidation of the compounds **5a**–**m** was accomplished using 1D and 2D-NMR spectroscopic and mass spectral analysis. The ^1^H-NMR spectrum of **5i** (Figure 2) has a doublet at δ 4.30 ppm (*J* = 10.0 Hz) for the benzylic proton 4′-CH of the pyrrolidine ring, which shows HMBCs with the spiro carbon C-3′ at 70.79 ppm and the methine carbon C-5′ at 61.09 ppm. The multiplet at δ 4.66–4.70 ppm was assigned to 5′-CH based on its H,H-COSY correlation with 4′-CH. Furthermore, the mutiplet at δ 2.70–2.75 and doublet of doublets 3.00–3.04 ppm (*J* = 14.5 Hz) were assigned to 6′-CH_2_ as it shows a H,H-COSY correlation with 5′-CH. The two doublets at δ 3.77 and 2.55 ppm (*J* = 14.0 Hz) can be assigned to the 2″-CH_2_ hydrogens as they show HMBCs with C-2′ at 67.12 and C-3′ at 70.79 ppm, besides a correlation with the piperidone carbonyl at 197.06 ppm. The arylmethylene proton 7″-CH appeared as a doublet at δ 5.00 ppm (*J* = 14.5 Hz) while 8″-CH was assigned to a doublet at δ 6.52 ppm (*J* = 15.0 Hz). The signal assignments are consistent with the HMBC correlations depicted in Figure 3. The fact that the H-4′ benzylic proton appeared as a doublet at δ 4.31 ppm (*J* = 11.0 Hz) clearly establishes the regiochemistry of the cycloaddition, since this signal would be a singlet for the other possible regiomers that might have arisen from the cyloaddition. In the ^13^C-NMR spectrum, the signals at 70.83 and 67.12 ppm were attributed to C-3′ and C-2′, respectively, while the signals at 39.48 ppm, 47.36 ppm and 52.81 ppm were assigned to the three methylene carbons (C-6′, C-2″ and C-6″). The signals at 179.83 ppm and 197.06 ppm were due to the oxindole and piperidone carbonyls, respectively. The assignments of C-2″, C-6′, C-6″, C-2′, C-3′, C-4′ and C-5′ were also confirmed from DEPT-135 experiments. Furthermore, the presence of a molecular ion peak at *m*/*z* = 739 (M^+^) in the mass spectrum of **5i** confirms the formation of the spiroheterocyclic hybrid. The structure of other spiropyrolidines was also assigned by similar straightforward considerations. Finally, the regio and stereochemistries of the spiropyrrolidines were confirmed by single X-ray crystallographic [23,24] analysis (Figure 4).

**Scheme 3:**
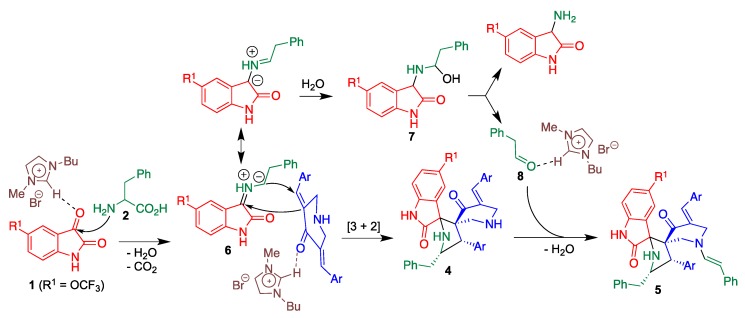
Plausible mechanism for the formation of spirooxindolopyrrolidine/*N*-styrylpiperidone heterocyclic hybrids in [bmim]Br.

A feasible mechanism proposed to rationalize the regio and stereoselective formation of compound **5** is summarized in Scheme 3. Initially, the interaction of [bmim]Br with the carbonyl group of 5-(trifluoromethoxy)isatin (**1**) via hydrogen bonding would increase the electrophilicity of the carbonyl carbon, facilitating the nucleophilic attack of the NH_2_ of l-phenylalanine (**2**) followed by dehydration and decarboxylation to furnish an azomethine ylide **6**. Similarly, the interaction of [bmim]Br with the carbonyl group of arylidenepiperidone presumably activates the exocyclic double bond, facilitating the addition of the azomethine ylide to the more electron deficient β-carbon of **3** to afford spiropyrrolidine **4**. Subsequently, the interaction of [bmim]Br with the carbonyl carbon of phenylacetaldehyde (**8**) would enhance its electrophilicity expediting the nucleophilic attack of the NH of piperidione reaction that affords an excellent yield of **5** via an enamine formation. Compound **8** would arise from the spontaneous decomposition of hemiaminal **7**, a product of the addition of water to the azomethine ylide **6**.

### 2.2. Biology

**Figure 5:**
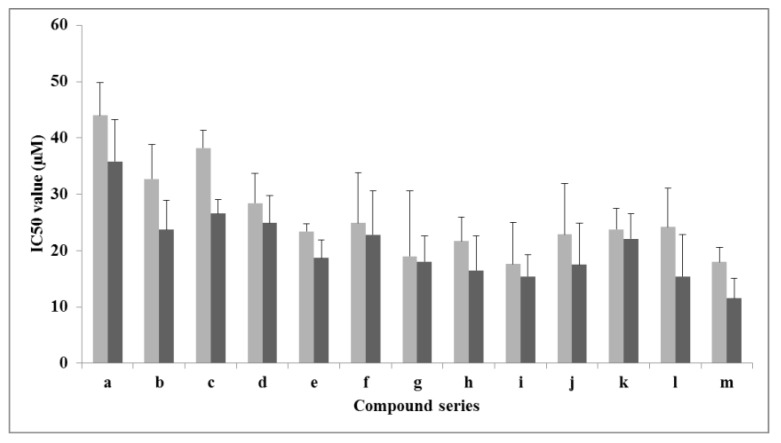
In vitro cytotoxicity assays for compounds **5a**–**m** against U-937 cell line for 24 h & 48 h. (data are mean ± SD of four replicates each). IC_50_ = concentration (µM) of drug required to inhibit growth of 50% of the cancer cells.

Compounds **5a**–**m** were evaluated for their cytotoxicity against the U-937 blood cancer cell line in comparison with the widely used drug bleomycin, using the MTT assay for 24 h and 48 h (Figure 5). The cytotoxicity effect of these compounds after 24 h follows the order (**5i** < **5m** < **5g** < **5h** < **5j** < **5e** < **5k** < **5l** < **5f** < **5d** < **5b** < **5c** < **5a**). The observed IC_50_ values (Figure 5) shown that compounds **5a**–**m** display a slightly higher cytotoxicity than the standard anticancer drug bleomycin (IC_50_ = 16.2 ± 4.5). The most active compounds were **5i** and **5m,** bearing *para*-methyl and *meta*-nitro group, respectively, on their substituted phenyl rings. Furthermore, compounds **5g** (*ortho*-methyl), **5h** (*meta*-methyl), **5j** (*ortho*-methoxy), **5k** (*meta*-methoxy) and **5l** (*para*-methoxy) exhibited good cytotoxicities. Among the compounds bearing halogen substituents on the phenyl ring, compound **4e** carrying a 2,4-dichloro substituent showed better activity than the others.

#### Quantification of Apoptotic Cell Percentage

**Figure 6:**
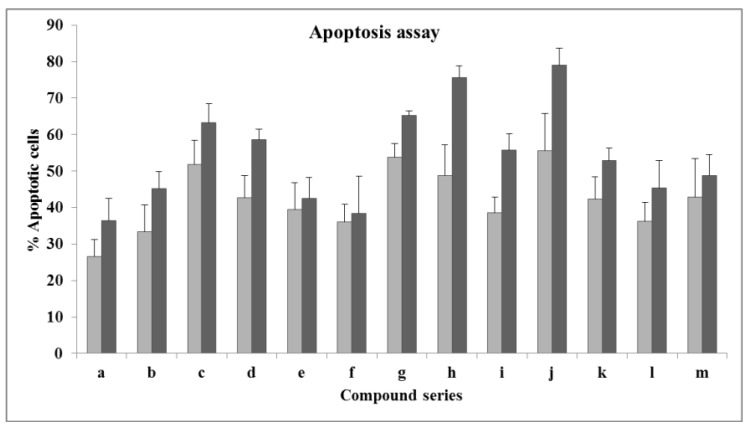
AO/EB morphological data showing the response of cells, in terms of apoptosis, to compounds **5a**–**m**. The percentage of cells in apoptosis is indicated by the histograms. The data shown are the means from triplicates. Vertical bars represent standard error of mean.

**Figure 7:**
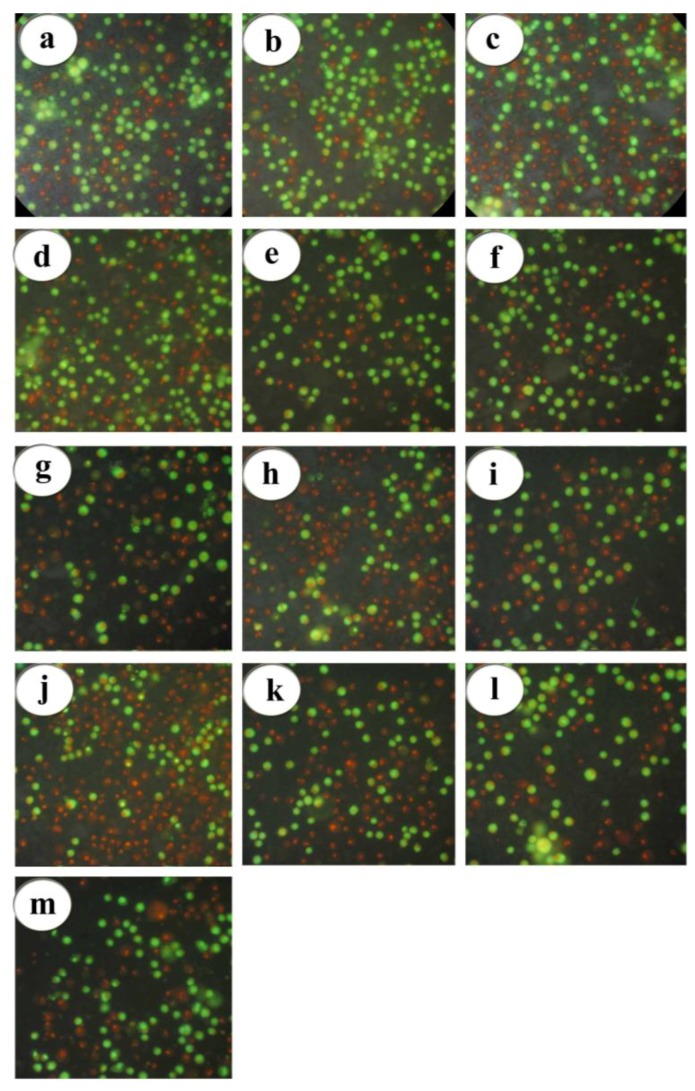
Cytological features of compounds **5a**–**m** treated U-937 cells (48 h).

**Figure 8:**
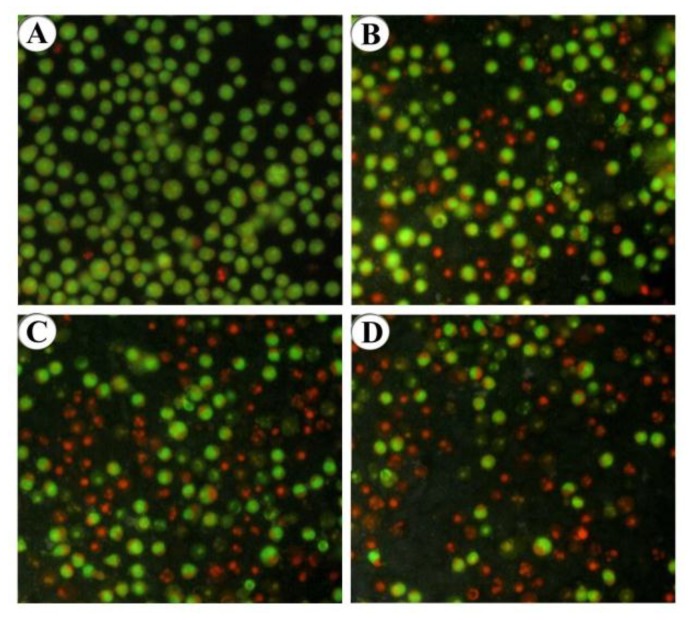
Photomicrographs of control (**A**) (the cells were viable as inferred from the green-fluorescence) and AO&EB stained U-937 cancer cells treated with compound (**5j**) (**B**)—dose IC_25_; (**C**)—IC_50_ and (**D**)—IC_75)_ for 24 h. 400×. In most of the cells with typical chromatin fragmented apoptotic cells.

**Figure 9:**
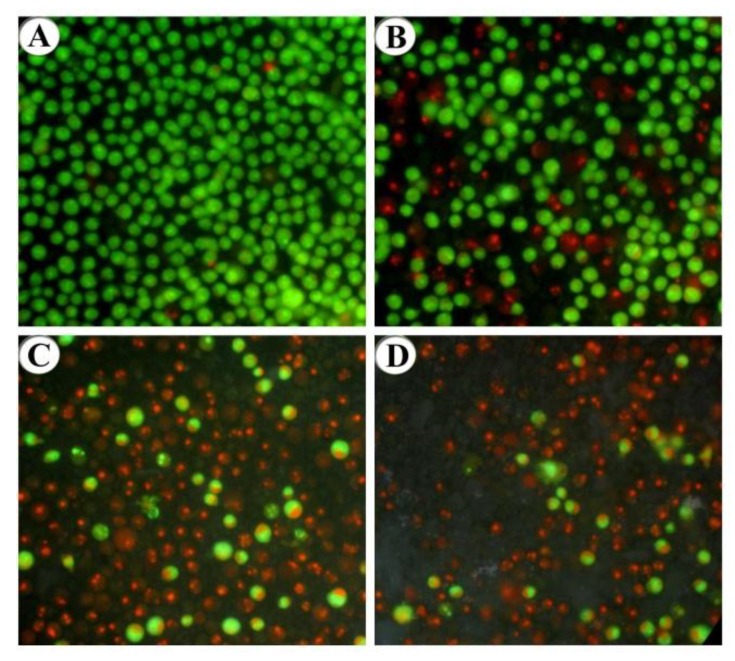
Photomicrographs of control (**A**) (the cells were viable as inferred from the green-fluorescence) and AO&EB stained U-937 cancer cells treated with compound (**5j**) (**B**)—dose IC_25_; (**C**)—IC_50_ and (**D**)—IC_75)_ for 48 h. 400×. In most of the cells with typical chromatin fragmented late apoptotic cells.

The morphology of the cells treated with the compounds **5a**–**m** has been used to determine the extent and nature of the cytological effects. A routine dual staining technique (AO & EB staining) was employed, which indicated the changes in the overall profile of the cell, with special reference to the cytoplasm and the nuclear morphology. AO & EB staining and fluorescence microscopy revealed apoptosis from the perspective of fluorescence (Figure 6). After the cells were exposed to IC_50_ concentrations of compounds **5a**–**m** for 24 h and 48 h, the cells were classified into four types according to the fluorescence emission and the nature of chromatin condensation in the nuclei (Figure 7) as follows: (i) viable cells had uniformly green fluorescing nuclei with a highly organized structure; (ii) early apoptotic cells (which still had intact membranes but had started undergoing DNA fragmentation) had green fluorescing nuclei, but peri-nuclear chromatin condensation was visible as bright green patches or fragments; (iii) late apoptotic cells had orange to red fluorescing nuclei with condensed or fragmented chromatin; and (iv) necrotic cells had uniformly orange to red fluorescing nuclei with no indication of chromatin fragmentation but the cells were swollen to a large size. The results indicated that treatment with the compounds **5a**–**m** caused more cells to take to apoptosis morphologies than necrotic features in dose and time-dependent manner. Considering apoptosis in isolation, its incidence was similar for all compounds. The data presented in Figure 6 and Figure 7 clearly show that the changes induced by the compounds **5a**–**m** were consistent with the induction of apoptotic cell death. Overall, the compounds under assay on U937 cells (**5j** > **5h** > **5g** > **5c** > **5d** > **5i** > **5k** > **5m** > **5l** > **5b** > **5e** > **5f** > **5a**) indicated a high incidence of apoptosis for 48 h, with the mode of cell death being dependent on the concentration and incubation time. For instance, AO&EB stained U-937 cancer cells treated with compound (**5j**) (b—dose IC_25_; c—IC_50_ and d—IC_75_) for 24 h and 48 h (Figure 8 and Figure 9). It was also apparent that the cells required a shorter incubation time for death only by apoptosis than by necrosis. In other words, the early response was death by apoptosis, and the cells that escaped this process succumbed to necrosis on short to prolonged treatment with compounds **5a**–**m**.

### 2.3. Molecular Docking

**Figure 10:**
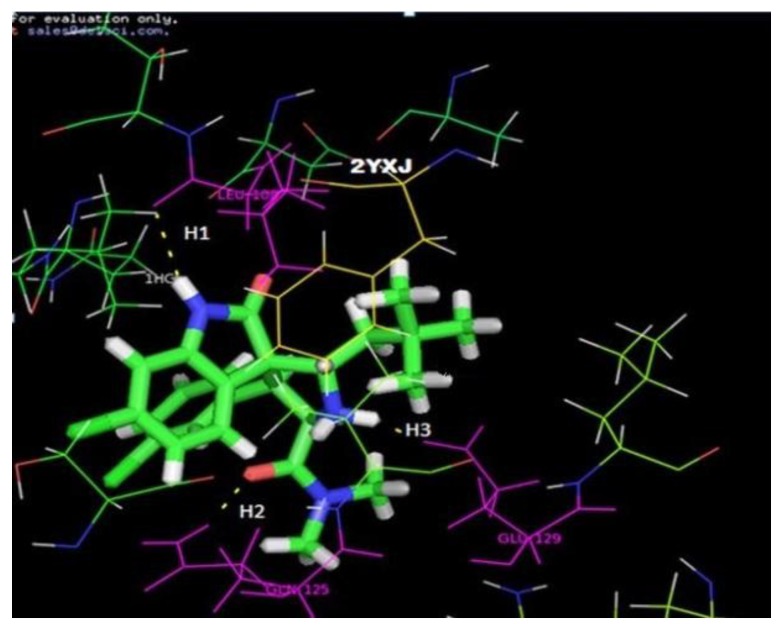
Actively docked conformation the compound (**5i**) into the receptor (2YXJ) cavity. The yellow colour dotted line represents the hydrogen bond interactions between the compounds (**5i**) into the receptor (2YXJ).

**Figure 11:**
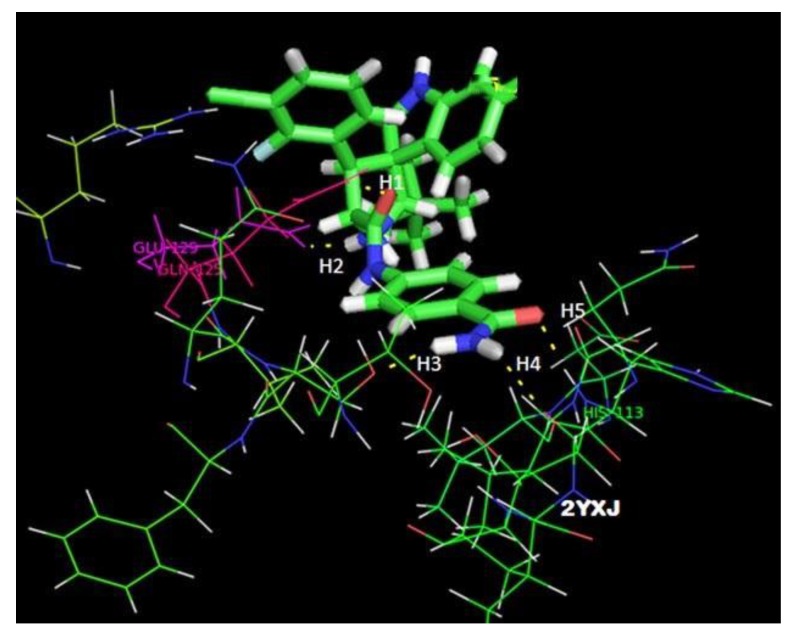
Actively docked conformation the compound (**5j**) into the receptor (2YXJ) cavity. The yellow colour dotted line represents the hydrogen bond interactions between the compound (**5j**) into the receptor (2YXJ).

Proteins belonging to the BCL-2 family (BCL-2, BCL-XL, and MCL1) are membrane proteins situated primarily on the outer membrane of mitochondria and are crucial to the apoptosis process [25]. Because some spiro-heterocyclic compounds related to ours have been shown to act by regulation of BCL-2 [26], we decided to undertake molecular docking analysis of the potential mode of action and binding efficiency between the ligands **5i** and **5j** and BCL-2 family proteins active sites. These compounds gave docking scores −16.0999 and −21.6782 (Table 2) and the docking structure of Figure 10 represents the hydrophobic interaction between **5i** and 2YXJ(Bcl-xl), with participation of the Gln125, Ser122, Glu129, Leu112, Leu108 and Val126 residues. Figure 11 represents the hydrophobic interaction between **5j** and 2YXJ with the participation of the Glu129, Leu112, Ser122, Val126 and Glu111 resudues. These results are compatible with a mechanism of action that involves the stimulation of the cancer cells death by inhibiting the Bcl-xL protein, particularly through programmed cell death (apoptosis), by regulating mitochondrial homeostasis [27].

## 3. Materials and Methods

### 3.1. General Information

^1^H, ^13^C and 2D NMR spectra were recorded on JEOL-400 and 500 MHz NMR spectrometer (Tokyo, Japan). Chemical shifts are given in parts per million (δ-scale) and the coupling constants are given in Hertz. Colum chromatography was performed on silica gel (60–120 mesh) using hexane:EtOAc as eluent. Single crystal X-Ray data set for 6 h was collected on Bruker APEXII D8 Venture diffractometer (Karlsruhe, Germany) with Mo Kα (λ = 0.71073 Å) radiation. Elemental analyses were performed on a Perkin Elmer 2400 Series II Elemental CHNS analyzer (Waltham, MA, USA).

### 3.2. General Procedure for Synthesis of Spiropyrrolidine Tethered Bis-Arylidenepiperidones ***5a***–***m***

A mixture of bisarylidenepiperidin-4-one (1.36 mmoL), isatin (2.72 mmoL) and l-phenylalanine (2.72 mmoL) were heated with stirring in [bmim]Br medium (3 mL) for 1 h at 100 °C. After completion of the reaction (TLC), ethyl acetate (2 × 5 mL) was added and the reaction mixture was stirred for 10 min. The organic layer was removed under reduced pressure and the crude product was purified by column chromatography (ethyl acetate: hexane *v*/*v* 3:7).

*5′-Benzyl-4′-phenyl-5-(trifluoromethoxy)spiro[3,2′]oxindolopyrrolidino-4′-phenyl-1”-styryl-5-benzylidene-spiro[3′.3″]piperidin-4″-one* (**5a**): Melting point 210–212 °C; white solid, 94%; ^1^H-NMR (CDCl_3_, 400 MHz): δ/ppm 5.00 (d, *J* = 14.64 Hz, 1H), 4.68–4.72 (m, 1H), 4.34 (d, *J* = 10.24 Hz, 1H), 3.80 (d, *J* = 13.92 Hz, 1H), 3.68 (d, *J* = 16.92 Hz, 1H), 3.47 (d, *J* = 5.4 Hz, 1H), 3.02 (d, *J* = 11.72 Hz, 1H), 2.72–2.77 (dd, *J* = 13.96, 8.08 Hz, 1H), 2.54 (d, *J* = 13.92 Hz, 1H), 6.56–6.63 (m, 2H), 6.92–7.42 (m, 23H, Ar); ^13^C-NMR (CDCl_3_, 100 MHz): δ/ppm 39.6, 47.3, 52.8, 53.1, 61.0, 67.3, 70.8, 100.9, 109.7, 121.1, 122.5, 124.1, 124.4, 126.4, 127.3, 128.5, 128.6, 128.7, 128.8, 129.3, 129.6, 130.1, 130.4, 134.5, 136.9, 138.3, 138.4, 138.7, 139.7, 139.8, 144.4, 179.8, 197.1. EI-MS: *m/z* 711 (M^+^). Anal. Calcd for C_44_H_36_F_3_N_3_O_3_: C, 74.25; H, 5.10; N, 5.90. Found: C, 74.38; H, 5.17; N, 5.81.

*5′-Benzyl-4′-(o-bromophenyl)-5-(trifluoromethoxy)spiro[3,2′]oxindolopyrrolidino-4′-(o-bromophenyl-1″-styryl-5-benzylidene-spiro[3′.3″]piperidin-4″-one* (**5b**): Melting point 247–249 °C; white solid, 96%; ^1^H- NMR (CDCl_3_, 400 MHz): δ/ppm 4.82 (d, *J* = 13.96 Hz, 1H), 4.58–4.61 (m, 1H), 4.49 (d, *J* = 10.24 Hz, 1H), 3.79 (d, *J* = 13.2 Hz, 1H), 3.60 (d, *J* = 16.82 Hz, 1H), 3.39 (d, *J* = 5.4 Hz, 1H), 2.94 (d, *J* = 13.96 Hz, 1H), 2.71–2.77 (m, 1H), 2.41 (d, *J* = 13.92 Hz, 1H), 6.56–6.61 (m, 2H), 6.98–7.65 (m, 21H, Ar); ^13^C-NMR (CDCl_3_, 100 MHz): δ/ppm 40.6, 47.4, 52.7, 53.2, 61.3, 67.2, 70.9, 100.8, 110.1, 121.9, 122.4, 122.5, 123.8, 124.5, 126.4, 126.5, 127.2, 128.3, 128.4, 128.5, 128.6, 128.7, 128.9, 129.1, 131.4, 131.6, 131.8, 133.2, 136.3, 138.2, 138.5, 138.6, 144.6, 179.5, 197.1. EI-MS: *m/z* 869 (M^+^). Anal. Calcd for C_44_H_34_Br_2_F_3_N_3_O_3_: C, 60.77; H, 3.94; N, 4.83; Found: C, 60.85; H, 3.83; N, 4.72.

*5′-Benzyl-4′-(p-bromophenyl)-5-(trifluoromethoxy)spiro[3,2′]oxindolopyrrolidino-4′-(p-bromophenyl-1″-styryl-5-benzylidene-spiro[3′.3″]piperidin-4″-one* (**5c**): Melting point 255–257 °C White solid, 97%; ^1^H- NMR (CDCl_3_, 400 MHz): δ/ppm 4.98 (d, *J* = 3.96 Hz, 1H), 4.56–4.62 (m, 1H), 4.22–4.29 (m, 1H), 3.74 (d, *J* = 13.2 Hz, 1H), 3.63 (d, *J* = 16.82 Hz, 1H), 3.37 (d, *J* = 15.4 Hz, 1H), 2.90 (d, *J* = 13.96 Hz, 1H), 2.72–2.78 (m, 1H), 2.53 (d, *J* = 13.92 Hz, 1H), 6.53–6.62 (m, 2H), 6.88–7.55 (m, 21H, Ar); ^13^C-NMR (CDCl_3_, 100 MHz): δ/ppm 39.7, 47.3, 52.8, 53.2, 61.4, 67.2, 70.8, 100.8, 110.0, 121.9, 122.4, 122.5, 123.8, 124.3, 126.4, 126.5, 128.5, 128.6, 129.1, 129.2, 130.7, 131.5, 131.7, 131.9, 133.2, 136.2, 136.3, 138.1, 138.4, 138.6, 140.2, 144.6, 179.6, 197.1. EI-MS: *m/z* 869 (M^+^). Anal. Calcd for C_44_H_34_Br_2_F_3_N_3_O_3_: C, 60.77; H, 3.94; N, 4.83; Found: C, 60.89; H, 3.81; N, 4.75.

*5′-Benzyl-4′-(o-chlorophenyl)-5-(trifluoromethoxy)spiro[3,2′]oxindolopyrrolidino-4′-(o-chlorophenyl-1″-styryl-5-benzylidene-spiro[3′.3″]piperidin-4″-one* (**5d**): Melting point 276–278 °C White solid, 93%; ^1^H- NMR (CDCl_3_, 400 MHz): δ/ppm 4.91 (d, *J* = 14.00 Hz, 1H), 4.61–4.68 (m, 1H), 4.20–4.27 (m, 1H), 3.87 (d, *J* = 13.2 Hz, 1H), 3.62 (d, *J* = 16.82 Hz, 1H), 3.35 (d, *J* = 15.4 Hz, 1H), 2.98 (d, *J* = 13.96 Hz, 1H), 2.77–2.84 (m, 1H), 2.45 (d, *J* = 13.92 Hz, 1H), 6.52–6.61 (m, 2H), 6.89–7.54 (m, 21H, Ar); ^13^C-NMR (CDCl_3_, 100 MHz): δ/ppm 40.5, 47.3, 51.9, 53.2, 62.9, 65.7, 73.2, 98.8, 110.1, 120.4, 122.5, 123.9, 124.1, 126.4, 126.5, 127.0, 128.0, 128.1, 128.2, 128.3, 128.4, 128.6, 128.9, 129.1, 129.7, 129.8, 130.5, 132.4, 132.9, 135.5, 136.3, 137.7, 138.8, 139.9, 144.4, 179.7, 198.9. EI-MS: *m/z* 780 (M^+^). Anal. Calcd for C_44_H_34_Cl_2_F_3_N_3_O_3_: C, 67.70; H, 4.39; N, 5.38; Found: C, 67.57; H, 4.47; N, 5.28.

*5′-Benzyl-4′-(o,p-dichlorophenyl)-5-(trifluoromethoxy)spiro[3,2′]oxindolopyrrolidino-4′-(o,p-dichlorophenyl-1″-styryl-5-benzylidene-spiro[3′.3″]piperidin-4″-one* (**5e**): Melting point 288–290 °C White solid, 94%; ^1^H- NMR (CDCl_3_, 400 MHz): δ/ppm 4.86 (d, *J* = 14.00 Hz, 1H), 4.68 (d, *J* = 13.92 Hz, 1H), 4.49–4.53 (m, 1H), 3.85 (d, *J* = 13.2 Hz, 1H), 3.58 (d, *J* = 16.82 Hz, 1H), 3.26 (d, *J* = 15.4 Hz, 1H), 2.95 (d, *J* = 13.96 Hz, 1H), 2.77–2.85 (m, 1H), 2.38 (d, *J* = 13.92 Hz, 1H), 6.49–6.59 (m, 2H), 6.00–7.76 (m, 19H, Ar); ^13^C-NMR (CDCl_3_, 100 MHz): δ/ppm 40.6, 46.3, 51.9, 52.3, 63.3, 65.5, 70.4, 99.3, 110.5, 121.8, 122.4, 123.9, 124.2, 126.4, 126.6, 126.5, 127.1, 128.4, 128.5, 128.6, 129.0, 129.2, 130.6, 132.4, 133.5, 134.2, 135.6, 135.8, 135.9, 136.8, 137.4, 138.1, 138.5, 132.9, 144.5, 177.8, 199.7. EI-MS: *m/z* 849 (M^+^). Anal. Calcd for C_44_H_32_Cl_4_F_3_N_3_O_3_: C, 62.21; H, 3.80; N, 4.95; Found: C, 62.36; H, 3.99; N, 4.83.

*5′-Benzyl-4′-(p-chlorophenyl)-5-(trifluoromethoxy)spiro[3,2′]oxindolopyrrolidino-4′-(p-chlorophenyl-1″-styryl-5-benzylidene-spiro[3′.3″]piperidin-4″-one* (**5f**): Melting point 259–261 °C; white solid, 95%; ^1^H- NMR (CDCl_3_, 400 MHz): δ/ppm 4.60 (d, *J* = 13.92 Hz, 1H), 4.18–4.23 (m, 1H), 3.86 (d, *J* = 10.28 Hz, 1H), 3.24–3.38 (m, 2H), 2.92–3.00 (m, 2H), 2.50–2.54 (m, 1H), 2.33–2.39 (m, 1H), 6.53–6.56 (m, 2H), 6.67–7.02 (m, 21H, Ar); ^13^C-NMR (CDCl_3_, 100 MHz): δ/ppm 39.8, 46.9, 52.3, 53.1, 61.5, 65.9, 70.7, 100.4, 109.8, 123.9, 124.1, 126.0, 128.1, 128.2, 128.4, 128.7, 128.8, 129.0, 130.4, 131.4, 132.5, 132.6, 135.8, 135.9, 136.4, 140.6, 143.7, 136.2, 137.6, 138.5, 139.9, 144.4, 179.1, 196.9. EI-MS: *m/z* 780 (M^+^). Anal. Calcd for C_44_H_34_Cl_2_F_3_N_3_O_3_: C, 67.70; H, 4.39; N, 5.38; Found: C, 67.59; H, 4.51; N, 5.25.

*5′-Benzyl-4′-(o-tolyl)-5-(trifluoromethoxy)spiro[3,2′]oxindolopyrrolidino-4′-(o-methylphenyl)-1″-styryl-5-benzylidene-spiro[3′.3″]piperidin-4″-one* (**5g**): Melting point 229–231 °C; white solid, 89%; ^1^H-NMR (CDCl_3_, 400 MHz): δ/ppm 4.96 (d, *J* = 14.5 Hz, 1H), 4.53–4.62 (m, 1H), 4.21 (d, *J* = 10.0 Hz, 1H), 3.64 (d, *J* = 14.00 Hz, 1H), 3.59 (d, *J* = 15.00 Hz, 1H), 3.37–3.42 (dd, *J* = 15 Hz, 1H), 2.98–3.02 (dd, *J* = 14.5 Hz, 1H), 2.66–2.71 (m, 1H), 2.48 (d, *J* = 14.0 Hz, 1H), 2.33 (s, 3H), 2.31 (s, 3H), 6.49–6.58 (m, 2H), 6.99–7.39 (m, 21H, Ar); ^13^C-NMR (CDCl_3_, 100 MHz): δ/ppm 21.1, 21.5, 39.5, 47.4, 52.8, 52.9, 61.1, 66.9, 70.8, 100.7, 109.6, 119.6, 120.9, 121.5, 122.4, 123.9, 124.2, 126.4, 128.4, 128.5, 129.1, 129.3, 129.35, 130.4, 131.6, 133.7, 135.2, 135.8, 136.2, 136.8, 138.2, 138.4, 138.7, 139.6, 139.8. 140.0, 144.3, 179.9, 197.1. EI-MS: *m/z* 739 (M^+^). Anal. Calcd for C_46_H_40_F_3_N_3_O_3_: C, 74.68; H, 5.45; N, 5.68. Found: C, 74.78; H, 5.51; N, 5.81.

*5′-Benzyl-4′-(m-tolyl)-5-(trifluoromethoxy)spiro[3,2′]oxindolopyrrolidino-4′-(methylphenyl)-1″-styryl-5-benzylidene-spiro[3′.3″]piperidin-4″-one* (**5h**): Melting point 234–236 °C; white solid, 88%; ^1^H-NMR (CDCl_3_, 400 MHz): δ/ppm 4.99 (d, *J* = 14.5 Hz, 1H), 4.63–4.68 (m, 1H), 4.34 (d, *J* = 10.0 Hz, 1H), 3.77 (d, *J* = 14.00 Hz, 1H), 3.68 (d, *J* = 15.00 Hz, 1H), 3.38–3.47 (dd, *J* = 15 Hz, 1H), 3.00–3.03 (dd, *J* = 14.5 Hz, 1H), 2.72-2.76 (m, 1H), 2.56 (d, *J* = 14.0 Hz, 1H), 2.33 (s, 3H), 2.27 (s, 3H), 6.60 (d, *J* = 14.5 Hz, 2H), 6.79–7.71 (m, 21H, Ar); ^13^C-NMR (CDCl_3_, 100 MHz): δ/ppm 21.1, 21.4, 39.4, 47.4, 52.8, 52.9, 61.1, 66.9, 70.8, 100.8, 109.5, 119.5, 120.9, 121.5, 122.4, 123.9, 124.3, 126.3, 128.4, 128.5, 128.6, 129.2, 129.4, 129.5, 130.5, 131.7, 133.6, 135.3, 136.2, 136.8, 138.3, 138.5, 138.8, 139.7, 139.8. 140.0, 144.3, 179.7, 197.1. EI-MS: *m/z* 739 (M^+^). Anal. Calcd for C_46_H_40_F_3_N_3_O_3_: C, 74.68; H, 5.45; N, 5.68. Found: C, 74.77; H, 5.55; N, 5.81.

*5′-Benzyl-4′-(p-tolyl)-5-(trifluoromethoxy)spiro[3,2′]oxindolopyrrolidino-4′-(methylphenyl)-1″-styryl-5-benzylidene-spiro[3′.3″]piperidin-4″-one* (**5i**): Melting point 222–224 °C; white solid, 95%; ^1^H-NMR (CDCl_3_, 400 MHz): δ/ppm 5.00 (d, *J* = 14.5 Hz, 1H), 4.66–4.70 (m, 1H), 4.30 (d, *J* = 10.0 Hz, 1H), 3.77 (d, *J* = 14.00 Hz, 1H), 3.67 (d, *J* = 15.00 Hz, 1H), 3.41–3.50 (dd, *J* = 15 Hz, 1H), 3.00–3.04 (dd, *J* = 14.5 Hz, 1H), 2.70–2.75 (m, 1H), 2.55 (d, *J* = 14.0 Hz, 1H), 2.35 (s, 3H), 2.32 (s, 3H), 6.52 (d, *J* = 14.5 Hz, 1H), 6.58–7.48 (m, 21H, Ar); ^13^C-NMR (CDCl_3_, 100 MHz): δ/ppm 21.1, 21.4, 39.5, 47.4, 52.8, 52.9, 61.1, 66.9, 70.8, 100.7, 109.6, 119.5, 120.9, 121.5, 122.3, 123.9, 124.3, 126.3, 128.4, 128.5, 129.2, 129.4, 129.38, 130.5, 131.6, 133.7, 136.9, 138.3, 138.4, 138.7, 139.6, 139.8. 140.0, 144.3, 179.8, 197.1. EI-MS: *m/z* 739 (M^+^). Anal. Calcd for C_46_H_40_F_3_N_3_O_3_: C, 74.68; H, 5.45; N, 5.68. Found: C, 74.76; H, 5.53; N, 5.79.

*5′-Benzyl-4′-(o-methoxyphenyl)-5-(trifluoromethoxy)spiro[3,2′]oxindolopyrrolidino-4′-(o-methoxyphenyl)-1″-styryl-5-benzylidene-spiro[3′.3″]piperidin-4″-one* (**5j**): Melting point 295–297 °C; White solid, 92%; ^1^H- NMR (CDCl_3_, 400 MHz): δ/ppm 5.00 (d, *J* = 14.64 Hz, 1H), 4.69–4.72 (m, 1H), 4.41 (d, *J* = 10.24 Hz, 1H), 3.87 (d, *J* = 13.92 Hz, 1H), 3.83 (s, 3H), 3.81 (s, 3H), 3.72 (d, *J* = 16.92 Hz, 1H), 3.55 (d, *J* = 15.4 Hz, 1H), 3.05 (d, *J* = 11.72 Hz, 1H), 2.84–2.90 (dd, *J* = 13.96, 8.08 Hz, 1H), 2.75 (d, *J* = 13.92 Hz, 1H), 6.62–6.68 (m, 2H), 6.99–7.58 (m, 21H, Ar); ^13^C-NMR (CDCl_3_, 100 MHz): δ/ppm 39.7, 47.4, 52.7, 53.3, 55.4, 55.5, 61.2, 67.4, 70.9, 100.9, 109.8, 114.1, 114.3, 121.3, 122.4, 124.6, 124.9, 126.5, 127.4, 128.6, 128.7, 128.6, 128.7, 129.4, 129.8, 130.2, 130.5, 131.7, 134.5, 136.8, 138.4, 138.5, 138.7, 139.8, 139.9, 140.2, 144.4, 179.3, 197.5. EI-MS: *m/z* 771 (M^+^). Anal. Calcd for C_46_H_40_F_3_N_3_O_5_: C, 71.58; H, 5.22; N, 5.44. Found: C, 71.69; H, 5.36; N, 5.58.

*5′-Benzyl-4′-(m-methoxyphenyl)-5-(trifluoromethoxy)spiro[3,2′]oxindolopyrrolidino-4′-(m-methoxyphenyl)-1″-styryl-5-benzylidene-spiro[3′.3″]piperidin-4″-one* (**5k**): Melting point 285–287 °C; white solid, 94%; ^1^H-NMR (CDCl_3_, 400 MHz): δ/ppm 4.99 (d, *J* = 14.64 Hz, 1H), 4.68-4.71 (m, 1H), 4.39 (d, *J* = 10.24 Hz, 1H), 3.85 (d, *J* = 13.92 Hz, 1H), 3.80 (s, 3H), 3.78 (s, 3H), 3.70 (d, *J* = 16.92 Hz, 1H), 3.53 (d, *J* = 15.4 Hz, 1H), 3.03 (d, *J* = 11.72 Hz, 1H), 2.79–2.86 (dd, *J* = 13.96, 8.08 Hz, 1H), 2.72 (d, *J* = 13.92 Hz, 1H), 6.60–6.67 (m, 2H), 7.13–7.63 (m, 21H, Ar); ^13^C-NMR (CDCl_3_, 100 MHz): δ/ppm 39.6, 47.3, 52.6, 53.3, 55.4, 55.5, 61.2, 67.4, 70.9, 100.8, 109.7, 114.2, 114.3, 121.5, 122.3, 122.4, 124.5, 124.6, 124.8, 126.5, 127.4, 128.6, 128.8, 128.5, 128.7, 129.4, 129.9, 130.2, 130.6, 134.6, 136.8, 138.4, 138.4, 138.7, 139.8, 139.7, 144.3, 179.4, 197.5. EI-MS: *m/z* 771 (M^+^). Anal. Calcd for C_46_H_40_F_3_N_3_O_5_: C, 71.58; H, 5.22; N, 5.44. Found: C, 71.66; H, 5.32; N, 5.55.

*5′-Benzyl-4′-(p-methoxyphenyl)-5-(trifluoromethoxy)spiro[3,2′]oxindolopyrrolidino-4′-(p-methoxyphenyl)-1″-styryl-5-benzylidene-spiro[3′.3″]piperidin-4″-one* (**5l**): Melting point 270–272 °C; White solid, 92%; ^1^H- NMR (CDCl_3_, 400 MHz): δ/ppm 5.00 (d, *J* = 14.64 Hz, 1H), 4.67–4.70 (m, 1H), 4.33 (d, *J* = 10.24 Hz, 1H), 3.86 (d, *J* = 13.92 Hz, 1H), 3.81 (s, 3H), 3.79 (s, 3H), 3.76 (d, *J* = 16.92 Hz, 1H), 3.47 (d, *J* = 15.4 Hz, 1H), 3.06 (d, *J* = 11.72 Hz, 1H), 2.75–2.79 (dd, *J* = 13.96, 8.08 Hz, 1H), 2.72 (d, *J* = 13.92 Hz, 1H), 6.59–6.66 (m, 2H), 7.14–7.63 (m, 21H, Ar); ^13^C-NMR (CDCl_3_, 100 MHz): δ/ppm 39.6, 47.3, 52.7, 53.1, 55.3, 55.4, 61.1, 67.3, 70.9, 100.8, 109.7, 114.1, 114.4, 121.1, 122.2, 124.7, 124.9, 126.5, 127.4, 128.5, 128.6, 128.7, 129.3, 129.9, 130.1, 130.4, 134.5, 136.9, 138.3, 138.5, 138.7, 139.8, 139.8, 144.4, 179.6, 197.2. EI-MS: *m/z* 771 (M^+^). Anal. Calcd for C_46_H_40_F_3_N_3_O_5_: C, 71.58; H, 5.22; N, 5.44. Found: C, 71.71; H, 5.34; N, 5.56.

*5′-Benzyl-4′-(m-nitrophenyl)-5-(trifluoromethoxy)spiro[3,2′]oxindolopyrrolidino-4′-(m-nitrophenyl)-1″-styryl-5-benzylidene-spiro[3′.3″]piperidin-4″-one* (**5l**): Melting point 296–298 °C; white solid, 94%; ^1^H- NMR (CDCl_3_, 400 MHz): δ/ppm 5.05 (d, *J* = 14.64 Hz, 1H), 4.75–4.77 (m, 1H), 4.30 (d, *J* = 10.24 Hz, 1H), 3.86 (d, *J* = 13.92 Hz, 1H), 3.83 (d, *J* = 16.92 Hz, 1H), 3.49 (d, *J* = 15.4 Hz, 1H), 3.10 (d, *J* = 11.72 Hz, 1H), 2.73–2.77 (dd, *J* = 13.96, 8.08 Hz, 1H), 2.74 (d, *J* = 13.92 Hz, 1H), 7.01–6.68 (m, 2H), 7.18–7.82 (m, 21H, Ar); ^13^C-NMR (CDCl_3_, 100 MHz): δ/ppm 40.6, 47.4, 52.1, 53.3, 62.8, 65.6, 70.9, 98.8, 110.2, 121.9, 122.4, 122.5, 112.5, 123.6, 124.4, 124.5, 126.4, 126.5, 126.7, 128.7, 128.8, 128.9, 129.2, 129.4, 129.9, 132.4, 132.9, 135.6, 136.3, 137.7, 138.7, 139.9, 144.4, 179.8, 198.7. EI-MS: *m/z* 801 (M^+^). Anal. Calcd for C_44_H_34_F_3_N_5_O_7_: C, 65.91; H, 4.27; N, 8.73; Found: C, 65.99; H, 4.39; N, 8.86.

### 3.3. Cytotoxicity Assay

The compounds **5a**–**m** were prepared as stock solutions, to suit the universal 96 well plate map, at different concentrations in µM, dissolved in DMSO (Sigma-Aldrich, St. Louis, MO, USA) or H_2_O. Working solutions were prepared in the culture medium and added to the wells 24 h after seeding of 5 × 10^3^ to 7 × 10^3^ cells per well in 200 μL of fresh culture medium. DMSO/H_2_O was used as the solvent control. Microscopic cytological changes were monitored and photographed following exposure to different concentrations of the compounds for 24 h or 48 h using an inverted microscope (Carl Zeiss, Jena, Germany). After the respective periods of treatment, plates were centrifuged and washed with fresh growth medium. Then, 20 μL of MTT solution (5 mg/mL in phosphate-buffered saline (PBS)) was added to each well, and the plates were wrapped with aluminum foil and incubated for 4 h at 37 °C. The purple formazan product was dissolved in 100 μL of 100% DMSO. The absorbance was monitored at 570 nm (measurement) and 630 nm (reference) using a 96 well plate reader (Bio-Rad, Hercules, CA, USA). Data were collected for four replicates each and used to calculate the median effect dose or concentration, i.e., IC_50_ using the Calcusyn software (Version 2, Biosoft Great Shelford Cambride GB-United Kingdome).

### 3.4. AO & EB Fluorescent Assay for the Assessment of Cell Death

Acridine orange and ethidium bromide staining was performed as described by Spector et al. [28] The cells (5 × 10^5^) were incubated with acridine orange and ethidium bromide solution (1 part each of 100 μg/mL acridine orange and ethidium bromide in PBS) and mixed gently, and examined in a fluorescent microscope (Carl Zeiss) using a UV filter (450–490 nm). Three hundred cells per sample were counted in duplicate for each dose point. Cells were scored as viable, apoptotic or necrotic, as judged from nuclear morphology and membrane integrity, and the respective percentages of apoptotic and necrotic cells were then calculated. The cells of interest were photographed.

There have been several approaches to detect cell death. In this study, double fluorescent staining of acridine orange (AO) and ethidium bromide (EB) has been used. Thus, the cells have classified into five types according to the fluorescence emission and the morphological feature of chromatin condensation in the stained nuclei: (i) viable cells have uniformly green fluorescing nuclei with a highly organized structure; (ii) early apoptotic cells have green fluorescing nuclei, but the peri-nuclear chromatin condensation has visible as bright green patches or fragments; (iii) late apoptotic cells have orange–red fluorescing nuclei with condensed or fragmented chromatin; (iv) necrotic cells have uniformly red nucleus and cytoplasm and (v) red and/or green stained cells with non-apoptotic morphological features i.e., fragmented nuclei with original cell morphology, vacuolated cytoplasm, cytoplasmic lesion, etc., have indicated that the terminal point in necro-apoptosis has reached. Fluorescent images of compounds 5**a**–**m** induced morphological features were observed using a high efficiency fluorescent microscope with apoptome fitted with time-lap and imaging facilities (Carl Zeiss) and analyzed using Axioscope and Image J Software (Version 1.36, Dresden, Germany). The data presented are representative of those obtained in at least three independent experiments conducted in triplicate.

## 4. Conclusions

We have developed an environmentally benign domino one-pot multi-component synthesis of spiroheterocyclic hybrids in [bmim]Br. This protocol involves a three component 1,3-dipolar cycloaddition and a concomitant enamine formation reaction that led to the stereo- and regioselective formation in excellent yields of novel structurally intriguing spirooxindolopyrrolidine-tethered *N*-styrylpiperidones **5a**–**m**. These compounds arose from a domino process that generated two C-C and two C-N bonds in a single operation, together with four adjacent stereogenic bonds that were formed with full diastereocontrol. The compounds thus synthesized were evaluated for their cytotoxicity effect against U-937 tumor cells and it was found that compounds **5i** and **5m** displayed an excellent cytotoxicity effect, which was comparable to that of the standard drug bleomycin. In addition, these compounds have been investigated for their apoptosis-inducing properties in the U-937 cancer cell model.

## Figures and Tables

**Table 1:** Solvent-optimization for the synthesis of dispiropyrrolidine/*N*-styrylpiperidone heterocyclic hybrid, **5i**.

Entry	Solvent	Time (h)	Yield (%)
1	Methanol	2	90
2	[bmim]OH	1.5	85
3	[bmim]BF_4_	1.5	88
4	[bmim]PF_6_	1.5	87
5	[bmim]Br	1	95

**Table 2:** Binding mode of the ligand-receptor interactions.

Entry	Ligand Structure ID	Protein Structure ID	Binding Affinity	Number of Hydrogen Bond Interaction	Number of Hydrophobic Interactions
1.	5i	2YXJ	−16.0999	3	Gln125,Ser122,Glu129,Leu112,Leu108,Val126
2.	5j	2YXJ	−21.6782	5	Glu129,Leu112,Ser122,Val 126,Glu 111

## Supplementary Materials

Supplementary File 1
