# Supplementary material for: Multicomponent Domino Synthesis, Anticancer Activity and Molecular Modeling Simulation of Complex Dispirooxindolopyrrolidines
Source: Molecules. 2018 May 5;23(5):1094. doi: 10.3390/molecules23051094 (PMC6100567; doi:10.3390/molecules23051094)
Supplement: Supplementary File 1 [file molecules-23-01094-s001.pdf]

Multicomponent domino synthesis, anticancer activity and molecular modeling simulation of complex dispirooxindolopyrrolidines

Natarajan Arumugam,[1]* Abdulrahman I. Almansour,[1] Raju Suresh Kumar,[1] Periyasami Govindasami,[1] Dhaifeallah Mohammed Qaide Althomili[1], Rajapandian Krishnamoorthy,[2] Vaiyapuri Subbarayan Periasamy,[3] Ali A. Alshatwi,[2] S. M. Mahalingam,[3] Shankar Thagamani,[4] J. Carlos Menéndez[5]

[1]Department of Chemistry, College of Science, King Saud University, P.O Box 2455, Riyadh 11451, Saudi Arabia; almansor@ksu.edu.sa (A.I.A); sraju@ksu.edu.sa (R.S.K); pkandhan@ksu.edu.sa (P.G); daife54321@hotmail.com

[2]Nanobiotecnology and Molecular Biology Research Laboratory, Department of Food Science and Nutrition, College of Food and Agricultural Sciences, King Saud University, Riyadh, Saudi Arabia; rjpandiyank@gmail.com (R.K); vsperrys@gmail.com (V.S.P); alshatwi@ksu.edu.sa (A.A.A)

[3]Purdue University, Department of Chemistry, 560 Oval Drive, West Lafayette, IN 47907-2084, USA; mahalins@purdue.edu (S.M.M)

[4]Department of Pathology and Population Medicine, College of Veterinary Medicine, Midwestern University, Glendale, Arizona 85308; sthang@midwestern.edu (T.S)

[5]Unidad de Química Orgánica y Farmacéutica, Departamento de Química en Ciencias Farmacéuticas, Facultad de Farmacia, Universidad Complutense, 28040 Madrid, Spain

Corresponding authors: anatarajan@ksu.edu.sa (N.A); josecm@farm.ucm.es(J.C.M)

S. No	List of Figures	Page No
1	Experimental and docking study details	S2
**2**	^{1}H NMR spectrum of **5i**	**S3**
**3**	Expanded ^{1}H NMR spectrum of **5i**	**S3**
**4**	^{13}C NMR spectrum of **5i**	**S4**
**5**	DEPT-135 spectrum of **5i**	**S4**
**6**	^{1}H,^{1}H-COSY spectrum of **5i**	**S5**
**7**	Expanded ^{1}H,^{1}H-COSY spectrum of **5i**	**S5**
**8**	^{13}C,^{1}H-COSY spectrum of **5i**	**S6**
**9**	HMBC spectrum of **5i**	**S6**
**10**	Mass spectrum of **5i**	**S7**

Experimental

General methods

^{1}H, ^{13}C and two-dimensional NMR spectra were recorded on a JEOL 400 and 500 MHz instrument in $CDCl_3$ using TMS as internal standard. Chemical shifts are given in parts per million (δ-scale) and the coupling constants are given in hertz. Elemental analyses were performed on a Perkin Elmer 2400 Series II Elemental CHNS analyser. Mass spectra were recorded on a Quattro Premier™ instrument (Micromass, Milford, USA) equipped with an electrospray ionization source (Zespray) coupled with an Acquity® UPLC system. Column chromatography was performed on silica gel (230-400mesh) using petroleum ethyl acetate as eluent.

Molecular docking

Molecular docking was performed using patch dock Geometry based algorithm server [23]. Structure of dispiropyrrolidine was drawn using ChemSketch integrated software package from Advance Chemistry and 3D optimization algorithm permits the planer (2D) structure from ChemSketch to promptly transform into an accurate 3D structure. It is constructed on the modified molecular mechanics which take into account, bond stretching, angle bending, internal rotation, and Vander Waals non bonded interaction. Protein preparation 3D X-ray crystal structures of ESBL protein (PDB ID - 4DTO) molecules was retrieved from Protein Data Bank (PDB) (www.rcsb.org).

The input file of both ESBL protein and dispiropyrrolindes was in the form of PDB format was submitted in patch dock server tool. The output file was as a docking report. The docked image was viewed by "Pymol 1.3" software. The interactions between ligands and proteins were also seen with the length of the interaction along with amino acids involved in these interactions and it was calculated by using "Pymol 1.3" software. Docking file of these poses was submitted to http://www.ebi.ac.uk/ pdbsum. Ligp lot of interactions of hydrophobic bonds between ligand and 4DTO were obtained.

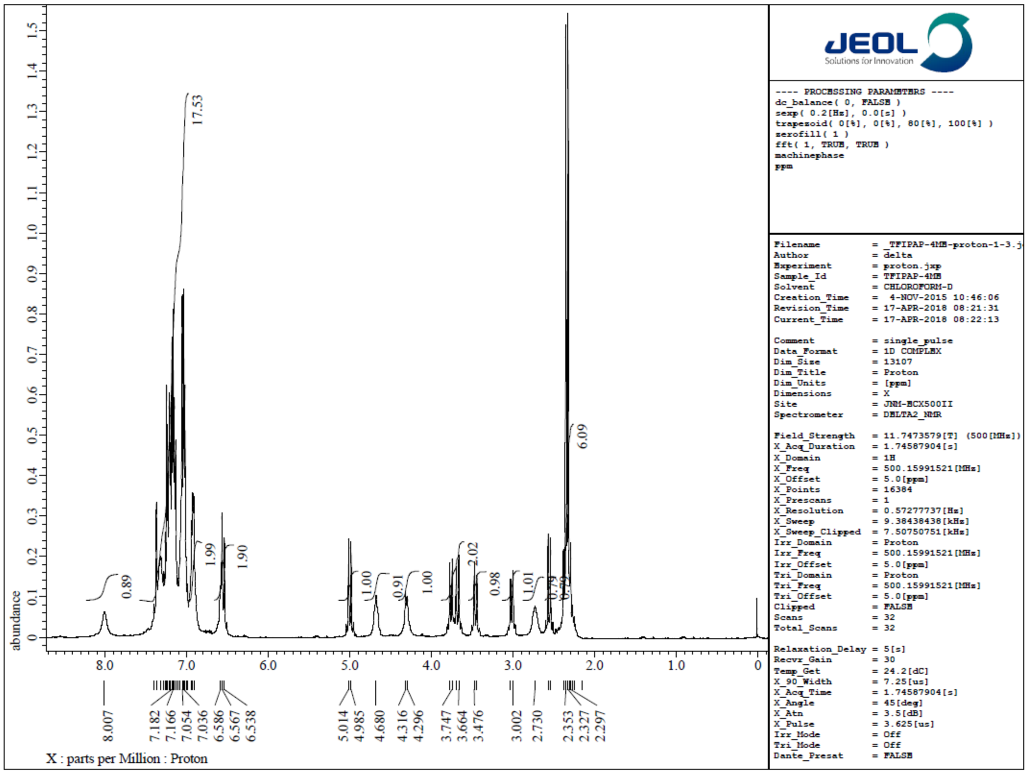

Figure 1. ^{1}H NMR spectrum of **5i**

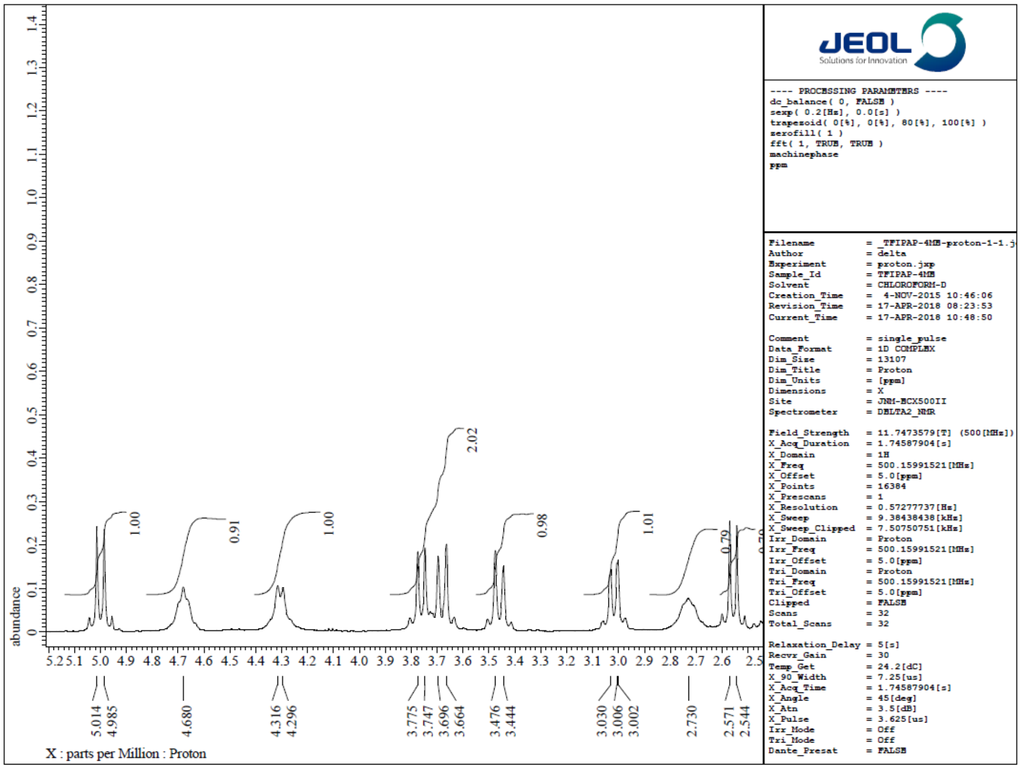

Figure 2. Expanded ^{1}H NMR spectrum of **5i**

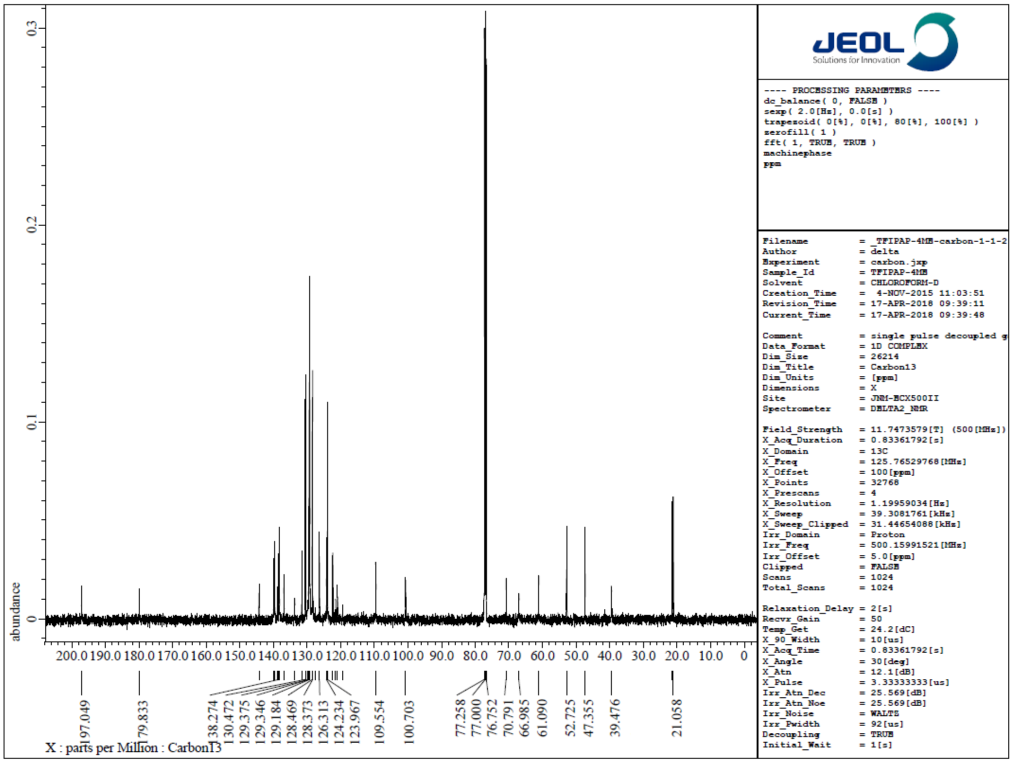

Figure 3. ^{13}C NMR spectrum of **5i**

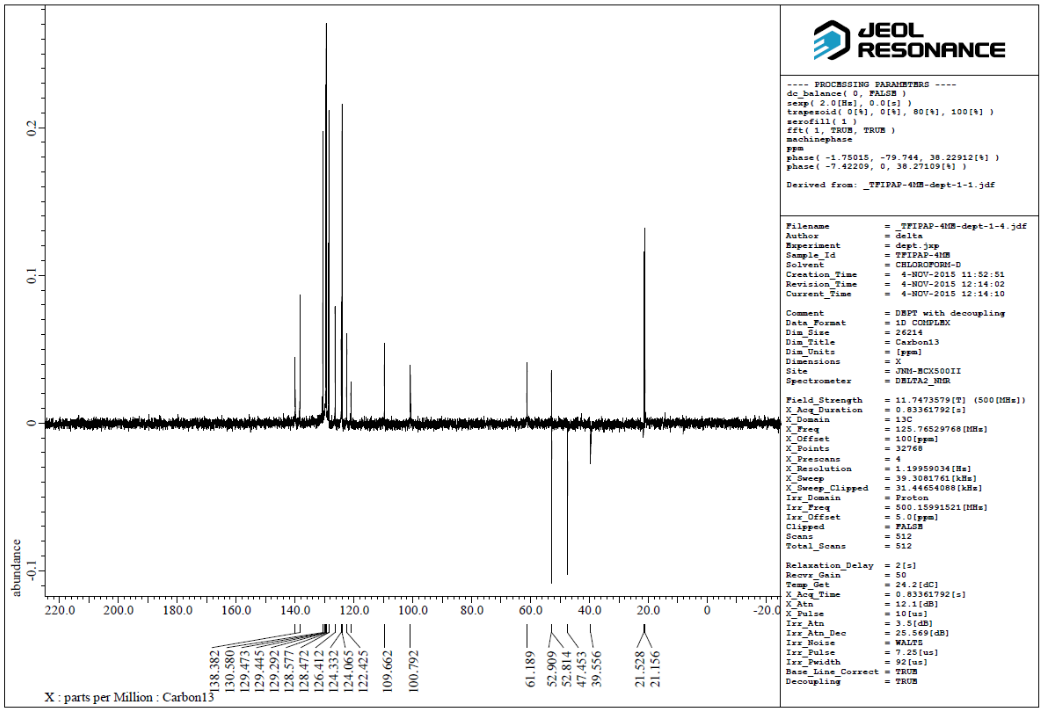

Figure 4. DEPT-135 spectrum of **5i**

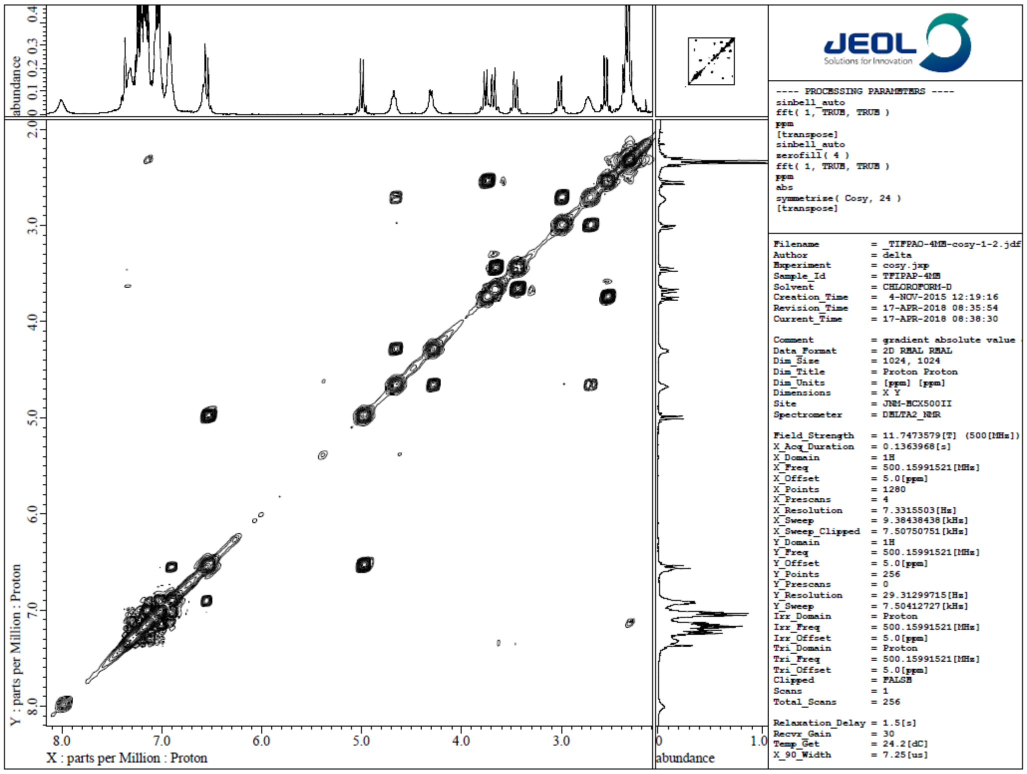

Figure 5. ^{1}H, ^{1}H-COSY spectrum of **5i**

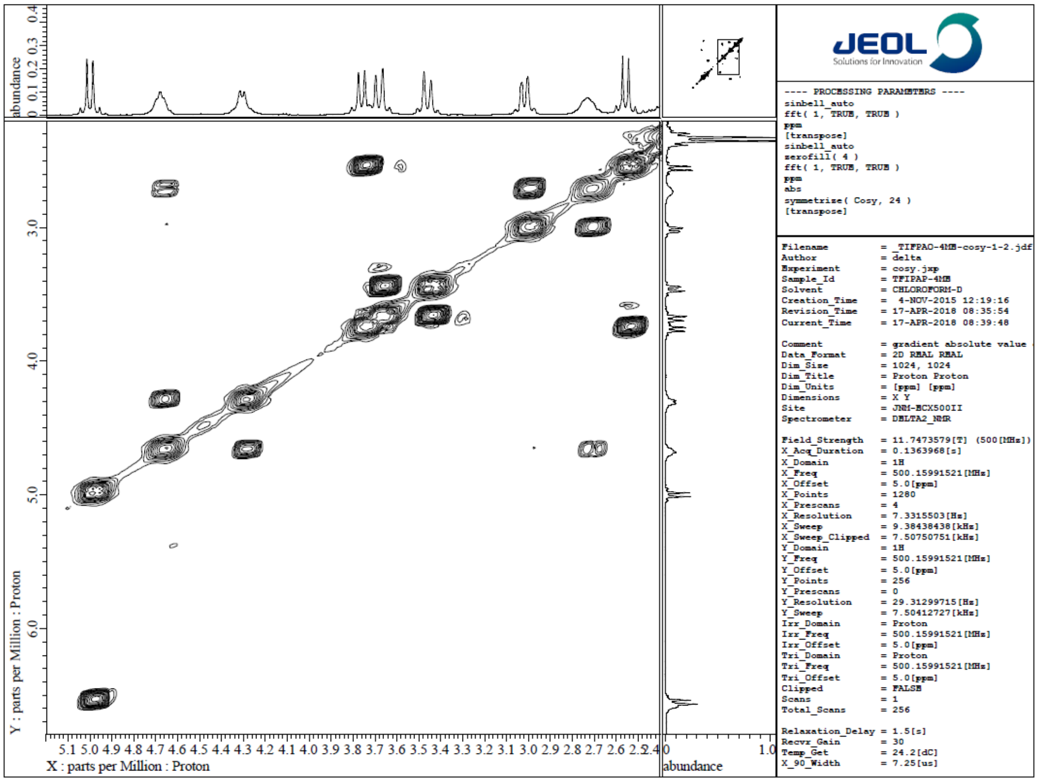

Figure 6. Expanded ^{1}H, ^{1}H-COSY spectrum of **5i**

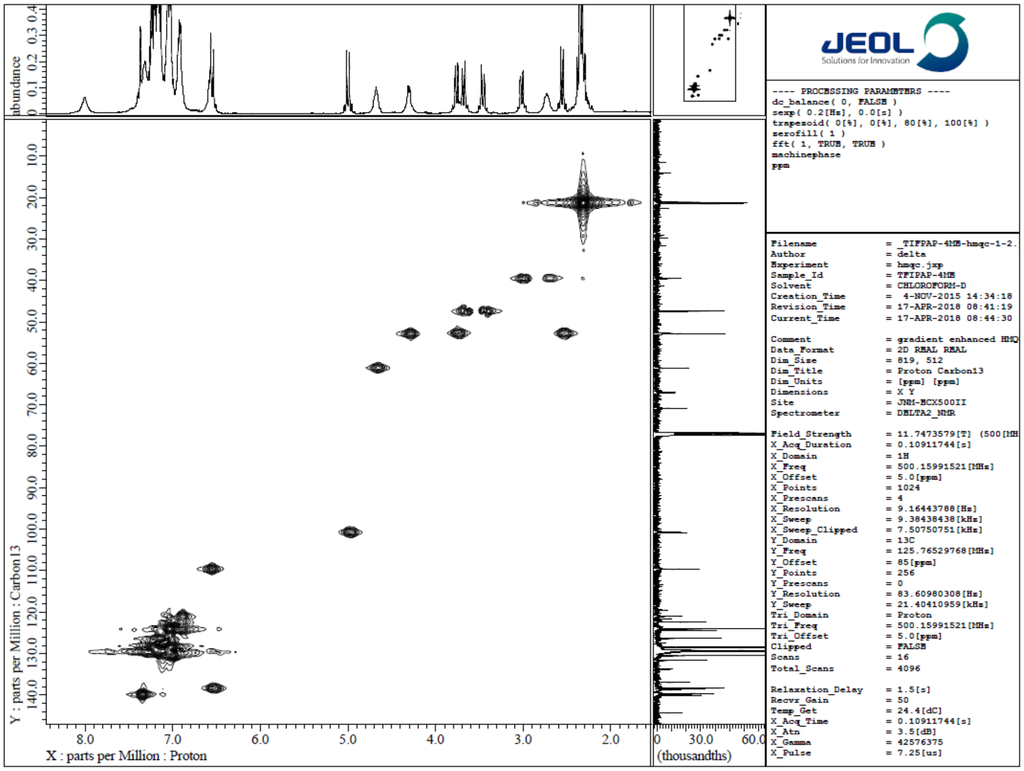

Figure 7. ^{13}C, ^{1}H-COSY (HMQC) spectrum of **5i**

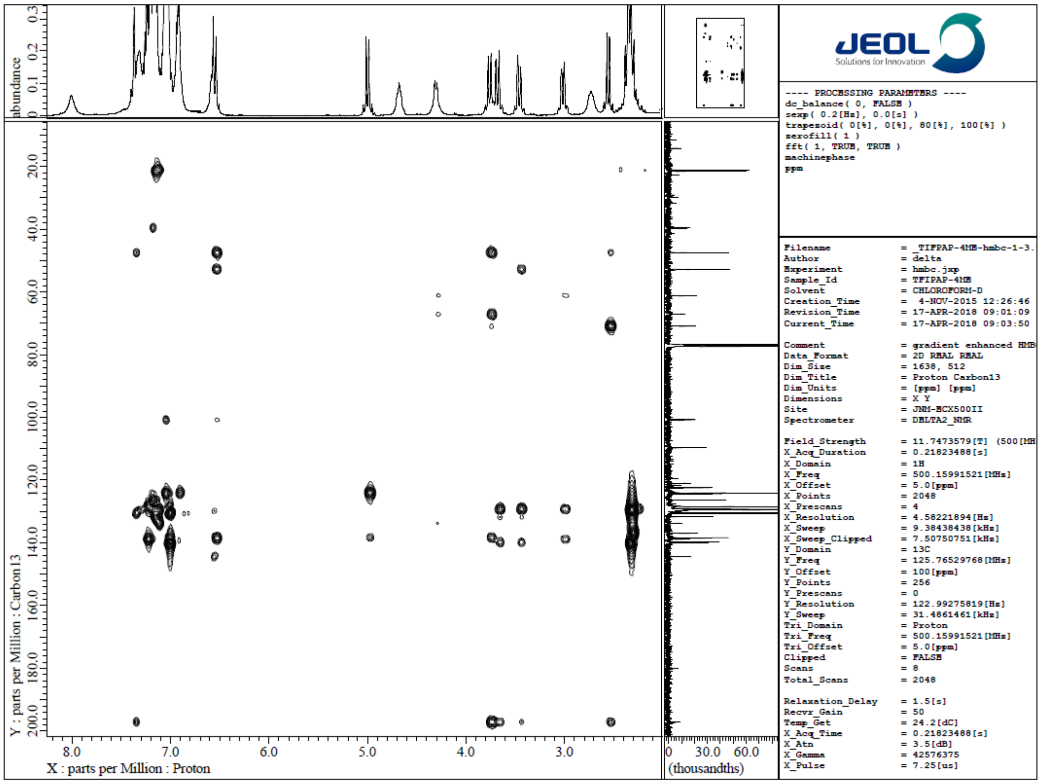

Figure 8. HMBC spectrum of **5i**

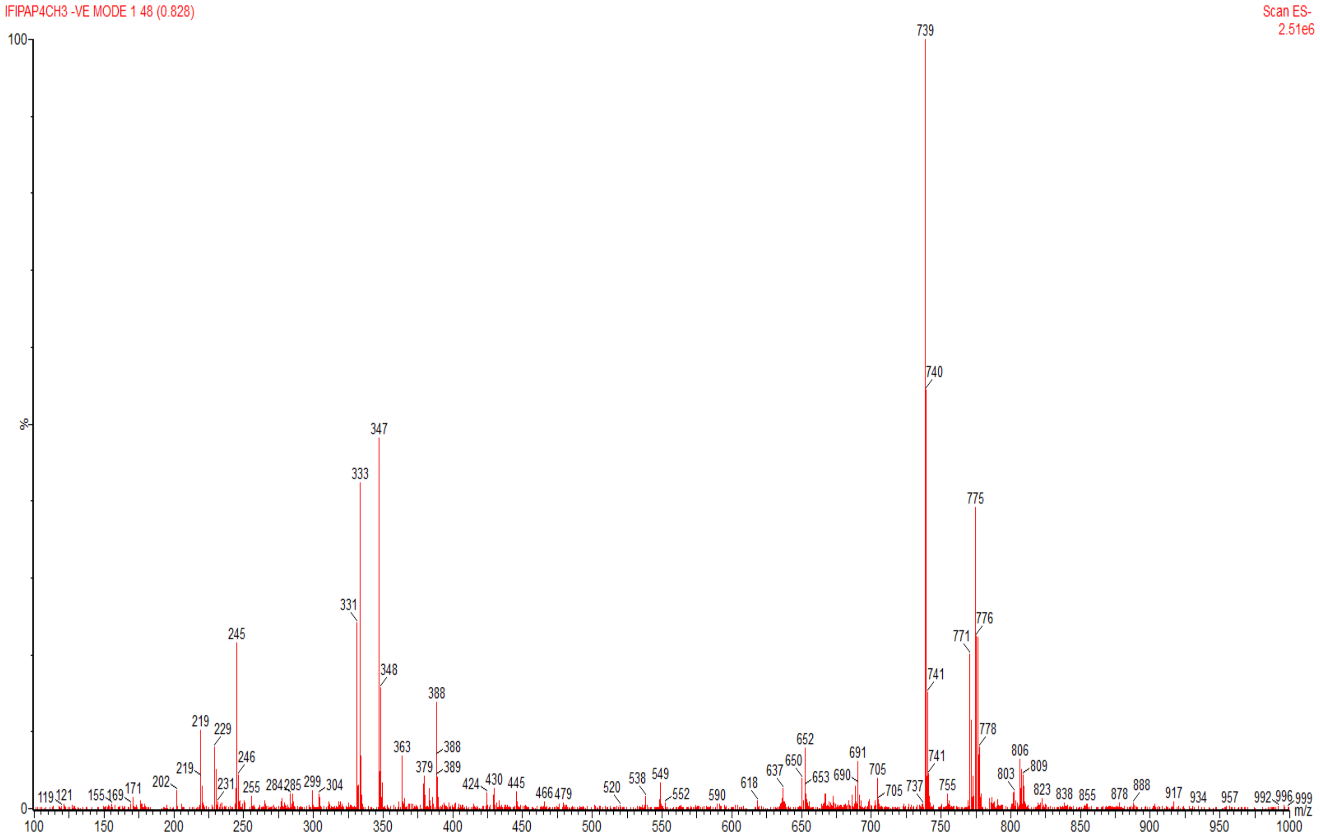

Figure 9. Mass spectrum of **5i**
